# Excavating the social representations and perceived barriers of organ donation in China over the past decade: A hybrid text analysis approach

**DOI:** 10.3389/fpubh.2022.998737

**Published:** 2022-09-26

**Authors:** Zizhong Zhang, Jing Jin, Chen Luo, Anfan Chen

**Affiliations:** ^1^School of Journalism and Communication, Tsinghua University, Beijing, China; ^2^School of Journalism and Communication, Wuhan University, Wuhan, China; ^3^School of Journalism and Communication, The Chinese University of Hong Kong, Hong Kong, Hong Kong SAR, China

**Keywords:** organ donation, social representation, China, semantic network analysis, conventional content analysis, cultural factors, social media

## Abstract

**Background:**

Organ donation has been claimed as a prosocial behavior to prolong the recipient's life and deliver great love. However, the supply-demand ratio of organs in China is highly unbalanced. Being entangled with multiple factors derived from individual and supra-individual levels, organ donation in China is important but sensitive. Previous scholars usually depended on obtrusive approaches to explore the facilitators and hindrances of organ donation, which is hard to discover genuine perceptions toward organ donation. Besides, relatively limited scholarly attention has been paid to what hampers organ donation in China.

**Objective:**

We intended to excavate the diversified social representations and perceived barriers to organ donation in China over the past decade.

**Method:**

Two kinds of text analysis methods—semantic network analysis and conventional content analysis, were applied to 120,172 posts from ordinary users on the Sina Weibo platform to address the research questions.

**Results:**

Regarding social representations, the “hope, understanding, and acceptance” of organ donation was the most pronounced one (34% of the whole semantic network), followed by “family story” (26%), “the procedure of organ donation in NGOs” (15%), “the practical value of organ donation” (14%), and “organ donation in the medical context” (11%). Regarding perceived barriers, a four-layer framework was constructed, including (1) the individual level, mainly about the fear of death and postmortem autopsy; (2) the familial level, which refers to the opposition from family members; (3) the societal level, which alludes to distrust toward medical institutions and the general society; (4) the cultural level, which covers religious-cultural concerns about fatalism.

**Conclusion:**

In concordance with prior works on social representations regarding organ donation, the current study also uncovered the coexistence of antithetical representations about organ donation—the longing for survival and the fear of death. This representation pair serves as the foundation of Chinese people's ambivalence. Besides, family-related narratives were dispersed over various representations, demonstrating the critical position of family support in organ donation. Moreover, the four-layer framework concerning donation barriers affords a reference for future empirical studies. The practical implications of this work are further discussed.

## Introduction

Organ donation is an important worldwide public health issue. Regrettably, many countries are facing a stagnant organ donation rate and an unbalanced organ supply-demand ratio (1), and China is no exception. According to official statistics, although the organ donation rate per million people has risen from 2.01 in 2015 to 4.16 in 2019 in China (2), the actual donation is far from meeting the demand (3). When it comes to the question of what hampers people from becoming potential organ donors, a considerable number of studies were conducted in Western contexts (4–6). While non-Western societies, like China, received scarce scholarly attention.

The current study aims to leverage social media traces to excavate how the Chinese public perceived organ donation (RQ1) and what constituted perceived barriers to organ donation (RQ2). RQ1 is analyzed in light of the social representation theory (SRT). SRT aims to disentangle people's daily meaning-making by analyzing opinions, knowledge, and beliefs around specific social objects (7). Previous researchers have drawn upon SRT to understand how people perceive organ donation (5, 8–11). For instance, Moloney et al. (9) found that organ donation-related representations include gifts of life, benefits to oneself, negative consequences, and concerns over medical care. Most of them were underpinned by two antithetical concepts—life and death. By adopting SRT, researchers can discover diverse public perceptions toward one issue and distill the essential driving factors behind the perceptions. Understanding public perceptions toward organ donation is of paramount importance in China. Different from countries applying the “opt-out” system (e.g., Chile, Spain), which means consent to posthumous organ donation is presumed unless citizens explicitly refuse (12), China implements an “opt-in” system, requiring manifested consent or authorization from organ donors or their immediate relatives (1). In this scenario, organ donation in China relies heavily on people's understanding and inner motivation. Therefore, comprehending the current perception landscape helps to find out the hidden cruxes and assists in tailored public health interventions to improve public knowledge and nurture a positive attitude about organ donation.

In terms of perceived barriers to organ donation, extant scholarship grounded in the Western context found that knowledge deficit, religious uncertainties, mistrust of medicine, hostility to new ideas, and misinformation were significant impediments to organ donation (13). By conducting a meta-synthesis of the qualitative literature regarding organ donation, Newton found that the desire to maintain an integral body and distrust in medical professionals are the most commonly identified barriers (14). Afifi et al. highlighted family resistance's adverse impact on becoming an organ donor (15). Another survey led by Stephenson et al. disclosed that the conception of bodily integrity had been a major deterrence of organ donation willingness (6). It is not hard to conjecture that barriers to organ donation are highly context-sensitive and can never be exhausted. In China, people's organ donation decisions may be intertwined with traditional spiritual beliefs (16), longstanding moral ethics (17), and other specific sociocultural factors (18). As one of the few empirical endeavors to supplement what makes the Chinese public reluctant to become organ donors, this study intends to respect the uniqueness of the Chinese context by extracting perceived barriers from voluntary disclosure more systematically.

Another point to be noted is that previous works regarding organ donation perception and perceived barriers were always situated in specific theoretical frameworks (15, 19, 20). On the one hand, established theories can shed light on comprehending organ donation succinctly and compactly; on the other hand, predefined theoretical frameworks limit motivators or impediments to specific concepts and somewhat sacrifice the richness of public opinion. The current work draws insights from emergent social media discussions (i.e., a corpus-driven approach) rather than the previously widely adopted theory-driven approach. Social media traces enable researchers to procure unobtrusive and naturalistic data from diverse audiences and help avoid social desirability bias that threatens traditional studies developed on survey data (21). We believe this exploratory research would extend the scholarship regarding Chinese people's perceptions of organ donation and contribute to pinpointing concerns when making the donation decision.

Specifically, this study adopts a hybrid text analysis approach to answer the two proposed research questions. The “hybrid” word means a combination of the quantitative and qualitative approaches in text analysis. The quantitative way emphasizes text as data, which aims to discover the underlying thematic or semantic structures of a given text (usually a large-scale corpus) by calculating the mathematical relationships among words. In contrast, the qualitative way mainly focuses on drawing insights by manual interpretation, which is more suitable for small-size data and an in-depth understanding of the implicit meaning. The following section will introduce the details of the hybrid text analysis approach and show how they help solve the two questions.

## Materials and methods

### Data source

Sina Weibo (hereafter referred to as Weibo) is a social media service launched in 2009. It has been acknowledged as the Chinese equivalent of Twitter. According to Weibo's annual report, the number of monthly active users has transcended 500 million as of September 2020 (22). Continuous content contribution from considerable active users makes Weibo an ideal platform for comprehending public perceptions or attitudes toward diverse issues (3, 23). Although the user characteristics of Weibo are not entirely chimed with the actual demographic characteristics in China, it remains an important window to understanding public opinion and social mentality.

After getting approval from the ethics committee in the first author's affiliation (No. THU202211), a Python web scraping script was developed to collect relevant posts from Weibo. According to the registration form for voluntary organ donation in China, we designated our search terms as “organ donation,” “donate organs,” “donate the body,” and combinations of the “donate” word with all types of organs listed in the form (see Supplementary material A for a bilingual inventory of search terms). Since Weibo launched its services at the end of 2009, we set our time range from January 1st, 2010 to December 31st, 2020.

**Figure 1:**
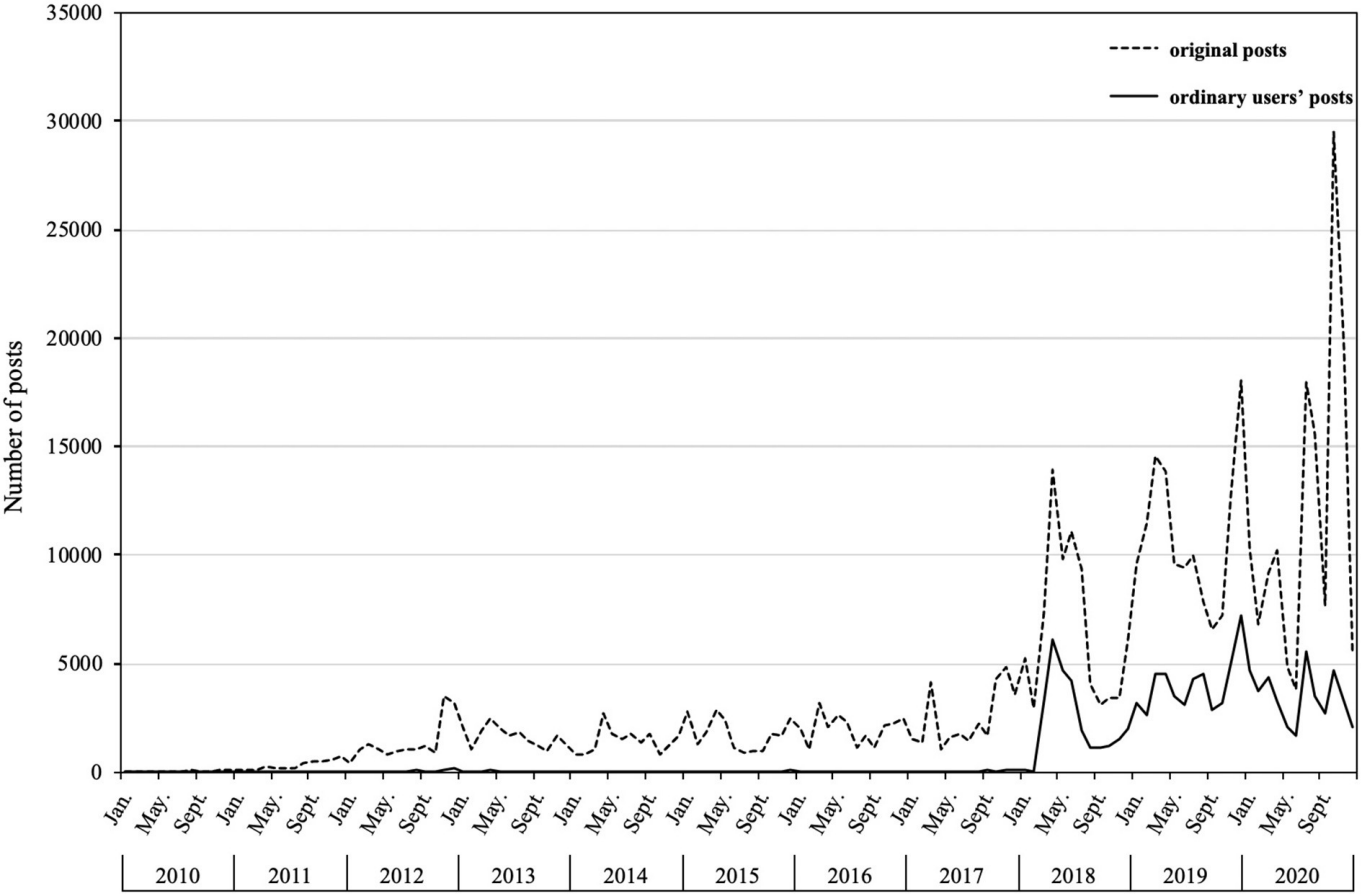
Distribution of the number of original and ordinary users' posts over time.

We pursued a fine-grained data collecting strategy by entering each search term into Weibo's advanced search platform and traversing through all conditions under each search option (e.g., time slot, location, user type). Simply put, we aimed to retrieve all posts in every subdivision of the combinations of search options. Each record in our dataset contains the Weibo content, user name, time of posting, user type, and other social media metrics. The original number of posts is 487,522. Since our focus is the public's social representations of organ donation, we excluded posts from accounts owned by governments, media, and other certified institutions. The quantity of ordinary users' posts is 120,172. Figure 1 illustrates the distribution of the number of original and ordinary users' posts over time. The two curves share a similar changing tendency.

### Methods

Following the hybrid approach, we choose appropriate methods according to the characteristics of the research questions. To address RQ1, semantic network analysis (SNA) was utilized to extract the major social representations around organ donation. Since social representations of a particular issue always reside in daily meaning-making, which manifests in diversified expressions, the considerable data volume calls for a highly efficient way to analyze the expressions automatically. SNA is a popular automated text analysis branch that demonstrates associations among concepts by discovering co-occurrence relationships (24, 25), which could further detect the most salient words and latent semantic structures from the text following a networked perspective (26). SNA has been widely adopted in social representation studies and coincides with the associative schema behind social representation's structural approach (27, 28). Compared with traditional manual coding, SNA is less affected by pre-defined theoretical rationale and opens to all possible semantically meaningful categories. These unique advantages make it an extensively used method in investigating online public opinion (29–31).

There are three requisite procedures in SNA. The first step is text preprocessing, including removing URLs, stopwords, punctuations, special characters, and search terms from the corpus. In the second step, we created the semantic matrix based on word co-occurrence. Miller suggested that people can only process an information unit with five to nine words at one time (32). Thus, we regarded two words as retaining a co-occurrence relationship if they appeared within a five-word chunk in one Weibo post (26, 31). We then calculated the co-occurrence frequency per word pair and filtered out word pairs below the average frequency (31). Lastly, Gephi, an open-source network analysis software, was leveraged for network visualization and clustering (33). We chose only the top 100 words by frequency for subsequent modularity analysis regarding our large corpus. In former studies, three essential indicators for network measurement—network density, degree centrality, and eigenvector centrality, were reported (26, 31, 34). Network density ranges from 0 to 1, referring to how intertwined the words are in a semantic network and the complexity of discussion around a particular issue. Mathematically speaking, network density equals the proportion of existing edges in all possible edges in an undirected network (35). Degree centrality represents the number of links one word has. In other words, it measures how many words are linked with the target word, which is intuitive and straightforward. Eigenvector centrality shows the centrality of one word in the network and indicates its relative influence. Generally speaking, a high eigenvector score denotes that a word is connected to many other words with high scores (36). Eigenvector centrality is based on degree centrality but is more advanced than degree centrality. Degree centrality or eigenvector centrality exhibits how pronounced a word is in a particular context. We also applied the LDA (Latent Dirichlet allocation) topic modeling to cross-validate the reliability of the SNA results. LDA topic modeling and SNA are widely adopted methods for identifying latent thematic structures in a given corpus (37). Different from SNA, LDA topic modeling is more algorithm-driven and relies on a three-level hierarchical Bayesian model to infer latent topics from recurring patterns of word occurrence in documents (37, 38).

Regarding RQ2, conventional content analysis, which is a typical method in the qualitative approach of text analysis, was employed. Hsieh and Shannon contended that conventional content analysis could be exploited to describe phenomena by directly extracting themes or labels from the text (39). In this way, scholars could immerse themselves in text and allow the emergence of new insights (40). Conventional content analysis is free from preconceived categories or theoretical frameworks (41). Conventional content analysis enables an in-depth comprehension of the corpus, through which researchers can build a conceptual framework based on subjective interpretation. 2,326 posts containing specific keywords were extracted from the corpus, including Chinese synonyms of the word “nonsupport” (i.e., “不理解”, “不支持”, “不选择”, “不接受”, “不同意” in Chinese) and the Chinese variations of the word “anxiety” (i.e., “担心”, “恐惧”, “害怕”, “忧虑”, “担忧”, “不安”, “顾忌”, “顾虑”, “怕” in Chinese). To ensure reliability and avoid biases that may be derived from manual interpretations, two coders first went through all selected posts and summarized several primary dimensions. Then 20% of the total posts (*n* = 465) were randomly sampled at the pilot stage for intercoder reliability assessment based on the preliminary dimensions. Cohen's Kappa was 0.93, indicating a satisfying agreement between the two coders according to previous practice (41). The remaining posts (*n* = 1,861) were split and coded by the two coders separately. Eventually, the coding results were merged into several meta categories.

In a nutshell, SNA was adopted on the whole corpus for extracting social representations automatically, and conventional content analysis was adopted on the selected posts about organ donation reactance or hesitance for a thorough understanding of perceived barriers. The two methods correspond to the quantitative approach and qualitative approach, respectively. However, they are complementary to each other for solving the research questions.

### Reliability and validity

Sufficient reliability and validity are necessary for the robustness of one study. Regarding the reliability of SNA, LDA topic modeling was employed to cross-validate the results of SNA. Specifically, after conducting a grid search for the best combination of prior parameters (e.g., the number of topics), the result of the best topic model acted as a standard of measurement of the SNA. A detailed comparison between the two approaches was exhibited in Supplementary material D. For the reliability of the conventional content analysis, previous experience showed that high intercoder reliability is a prerequisite for a reliable content analysis study (42). The value of Cohen's Kappa in our research indicates a nearly perfect agreement between the two coders (43), confirming the reliability of the content analysis part.

With respect to validity, compared to previous works, the current study is based on social media data across 11 years, which enables a higher probability of discovering diverse and comprehensive perceptions than those built on data within a limited time span. In other words, the internal validity of this study has gotten certain assurance. Regarding external validity or the generalizability of results, scholars contend that social media data are rarely representative and face the threat of biased structures (44, 45). The data volume can be neither treated as a sign of validity nor invalidity of the findings (45). This uncertainty is nearly an intrinsic shortcoming of social media studies and is hard to overcome at the present stage; thus, we proposed the limitation at the end of our study.

## Results

### Semantic network analysis

After time slicing, we found no significant variations in social representations over the past 11 years. Therefore, this study did not differentiate years when constructing the semantic network (please refer to Supplementary material B for semantic network and social representations summary per year). As mentioned before, only the top 100 words by frequency were incorporated in the final network, with 4,693 edges connecting them. The average degree of the network was 93.86, and the average weighted degree was 9,366. A network density value of 0.948 indicated that the network is relatively compactly interconnected. Table 1 exhibits the 30 most central terms along with their frequency, degree, weighted degree, and eigenvector centrality. The leading central words about organ donation on Weibo include *being* (*being* in this study means conscious existence or a living thing), *society, China, hope*, and *human organ*.

**Table 1:** Summary output of the top 30 central terms in the semantic network.

**No**.	**Term**	**Frequency**	**Degree**	**Weighted degree**	**Eigenvector centrality**
1	Being	27,007	99.00	51,545	1.00
2	Society	22,714	98.00	9,622	0.99
3	China	20,020	99.00	31,574	1.00
4	Hope	19,886	99.00	38,153	1.00
5	Human organ	17,805	99.00	31,102	1.00
6	Register	13,502	98.00	19,974	0.99
7	Passed away	12,611	99.00	28,423	1.00
8	World	12,035	99.00	18,518	1.00
9	Mom	11,768	97.00	17,233	0.98
10	Parents	9,199	98.00	20,412	0.99
11	Doctor	9,058	97.00	12,448	0.98
12	Volunteer	8,963	97.00	14,160	0.98
13	Child	8,846	99.00	14,640	1.00
14	Hospital	8,608	98.00	11,725	0.99
15	Families	7,623	99.00	15,558	1.00
16	Patient	7,467	97.00	18,343	0.98
17	Death	6,774	98.00	8,617	0.99
18	Girl	6,755	92.00	11,368	0.93
19	Continue	6,370	99.00	17,766	1.00
20	Son	6,248	98.00	14,023	0.99
21	Pass away	6,159	94.00	15,611	0.95
22	Before death	5,801	92.00	11,651	0.93
23	Living	5,729	98.00	7,644	0.99
24	Significance	5,725	98.00	10,007	0.99
25	Daughter	5,668	99.00	10,081	1.00
26	Body	5,562	97.00	8,901	0.98
27	Volunteering	5,525	94.00	8,675	0.95
28	Brain death	5,192	92.00	12,293	0.93
29	Life	5,163	98.00	6,804	0.99
30	Father	5,132	95.00	11,331	0.96

**Figure 2:**
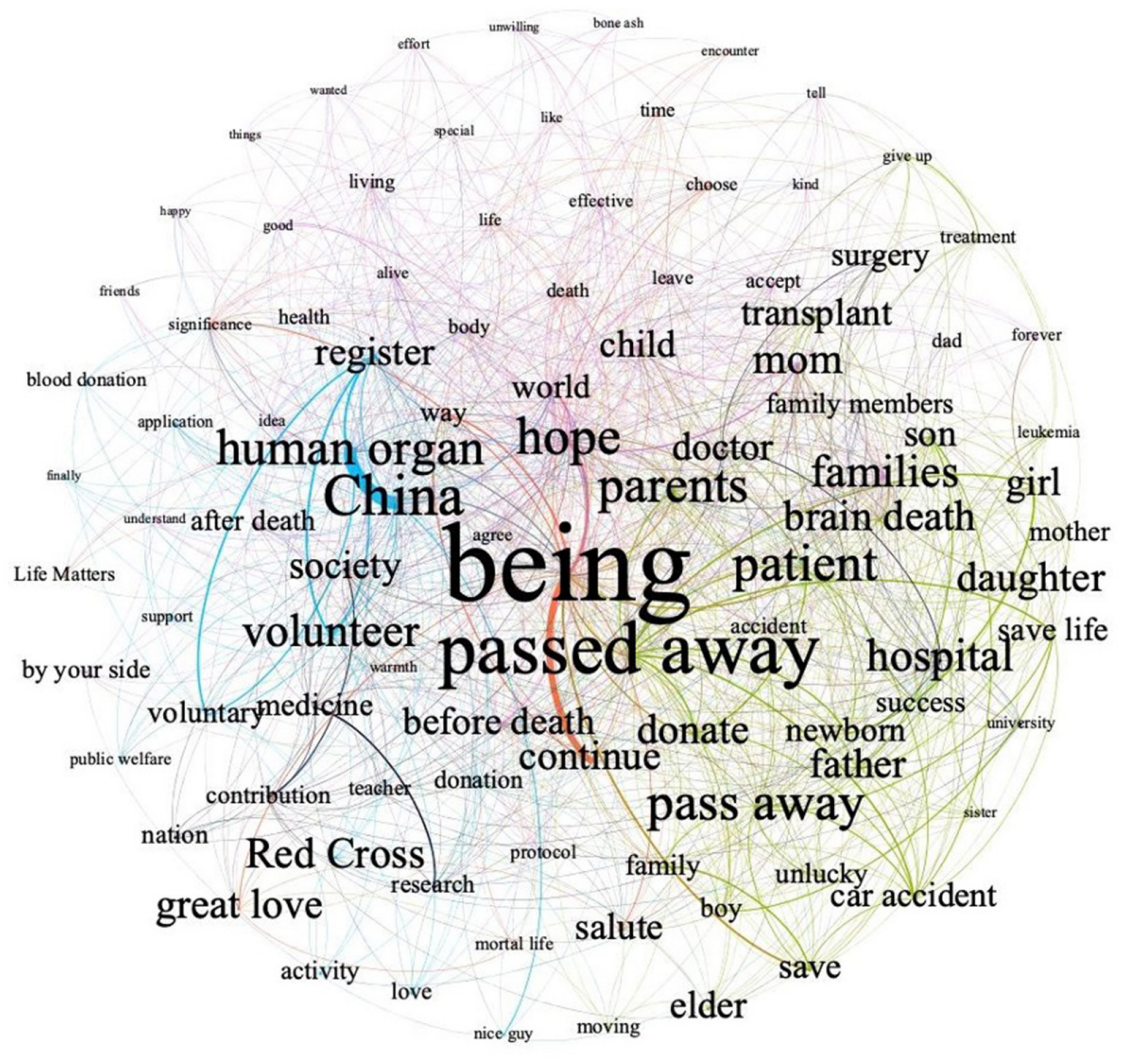
Semantic network visualization regarding organ donation on Weibo.

The semantic network is illustrated in Figure 2. The network's layout follows the ForceAtlas2 algorithm in Gephi. This layout algorithm performs better on convergence and compactness and gives nodes with a high degree centrality a more central position in the graph (46). For clarity, only edges with a weight above the average edge weight were exhibited (31). The entire semantic network was presented in Supplementary material C. Edges represent co-occurrence relationships between words, and their thickness embodies the co-occurrence frequency. The larger the word, the higher eigenvector centrality the word has, which manifests a more salient position of the corresponding word. Next, modularity analysis was conducted for community detection, which helps uncover the semantic substructures of a given network (47). With the assistance of modularity analysis, we extracted social representations of organ donation and rendered different colors to distinguish them. Table 2 enumerates all representations with their related terms and proportions.

**Table 2:** Summary of social representations derived from the semantic network.

**No**.	**Social representations**	**Top associations**	**Association count**	**Color**	**Share of the network (%)**
1	Hope, understanding, and acceptance	World-hope	1,853	Purple	34%
		Hope-before death	808		
		Hope-body	563		
		Agree-families	366		
		Hope-understand	363		
2	Family story	Family-save	1,600	Green	26%
		Save life-boy	1,290		
		Pass away-parents	1,237		
		Son-father	1,220		
		Son-car accident	1,129		
3	The procedure of organ donation in NGOs	China-human organ	12,359	Blue	15%
		Human organ-register	2,300		
		Volunteer-register	2,083		
		Human organ-volunteer	1,741		
		China-good guy	955		
4	The practical value of organ donation	Continue-being	6,428	Orange	14%
		Way-being	1,745		
		Significance-being	1,242		
		Great love-being	998		
		Being-salute	961		
5	Organ donation in the medical context	Medicine-research	1,724	Black	11%
		Society-contribution	1,152		
		Doctor-hospital	956		
		Medicine-contribution	676		
		Doctor-surgery	673		

Five social representations were drawn out from Figure 2, which objectifies prime public perceptions of organ donation on Chinese social media. The largest category was “hope, understanding, and acceptance” (34% of the network), mainly referring to the transmission of hope by donating organs and the reconciliation between organ donation and traditional beliefs. Hope contains two-fold meanings—one is about the motivation to donate organs, such as “I hope that after talking with my parents, I can sign the organ donation form.” The other is related to the expectation of new life, such as “When I leave this world in the future, at least four families can get hope again with my donated organs.” The second-largest category was “family story” (26% of the network), mainly about the experience of post-mortem organ donation of a family member, such as “There is a cute little kid in my neighborhood who passed away and his parents donated his organs. The child stayed alive in another way, and his parents are brave and kind.” “organ donation procedures in NGOs” (15% of the network) followed as the third-largest category, associated with specific procedures within the NGO system, such as registering as an organ donation volunteer and submitting the donation form. The fourth category revolved around “the practical value of organ donation” (14% of the network), emphasizing organ donation's merits in extending recipients' life length and improving recipients' life quality. Also, some users lauded organ donation as a manifestation of altruism and a noble deed of spreading great love. The last category discussed “organ donation in the medical context” (11% of the network), which focused on the importance of organ donation for medical experiments, research, and teaching.

Topic modeling based on the LDA algorithm was introduced to ensure our findings' robustness and reliability. Borrowing previous experience (37), we performed a grid search for the most reliable parameter combination. The semantic coherence value suggested a topic number of 5 is the most appropriate. All themes generated by the topic model are consistent with the modularity analysis results, lending credence to our findings' reliability. Please turn to Supplementary material D for the parameters selection process and the final output of the best-performing topic model.

### Conventional content analysis

**Figure 3:**
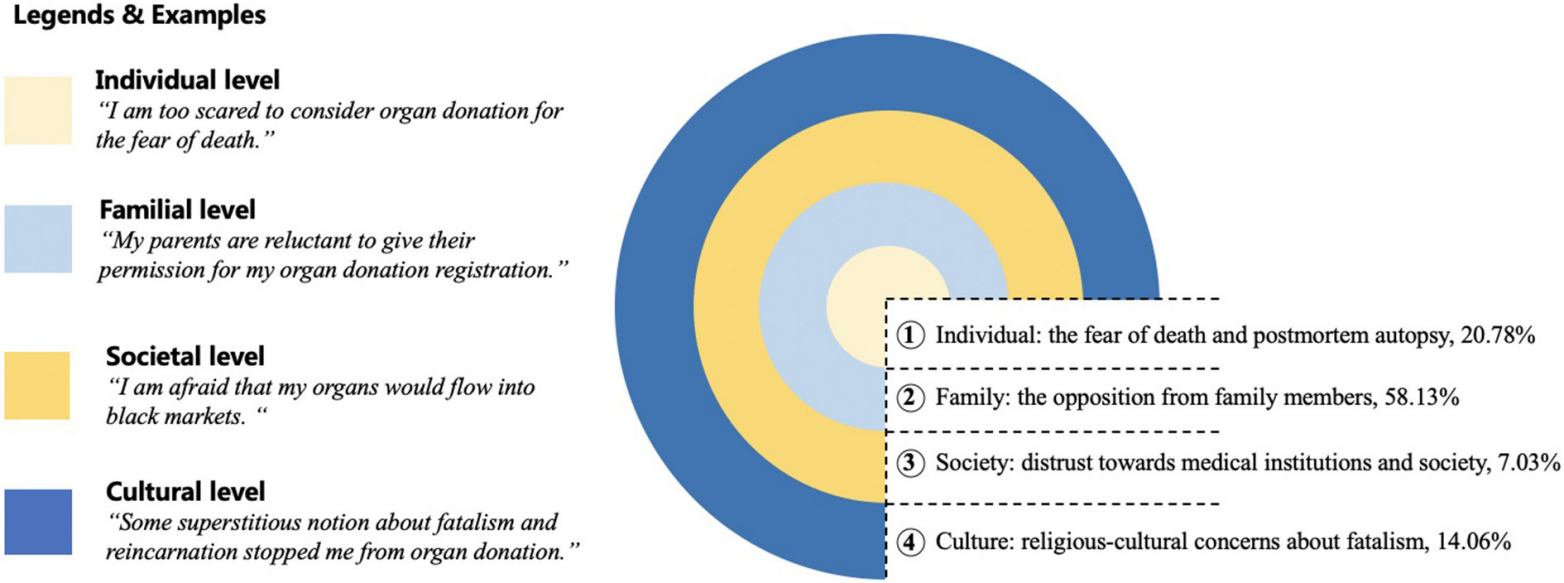
Hindrances to organ donation derived from the conventional content analysis.

We manually coded 2,326 posts containing the appointed keywords. The final classification framework was settled when the two coders found no new category emerged. In other words, we stopped adding new categories when the final framework reached saturation. The ultimate framework consists of four dimensions: individual perception, family disapproval, social mistrust, and cultural beliefs. Figure 3 depicts the four salient dimensions, with typical examples chosen from our corpus.

#### Individual level: The fear of death and postmortem autopsy

20.78% of the filtered posts demonstrated that hindrances to organ donation arose from individuals' dread of death, resistance to body exposure, fear of body dissociation. Those inhibitors also chime with the viewpoint of Moloney et al. that the fundamental representations of organ donation can't be separated from life and death (27). When encountering death-related topics like organ donation, individuals' survival instinct surpasses their rational judgments and fortifies the psychological discomfort of death. Sample posts are as follows.

*Organ donation and hospice care are callous things. It is so heavy to face death or organ donation. Everyone has a fear of death and what we should do is cherish life*.

*I was even naive enough to think about signing up for a body donation. And I'm afraid that my body will become a part of someone I have no connection to*.

The preoccupation with the body also hinders the willingness to donate organs due to the individual body perception (48). There exist two aspects of body perception regarding organ donation—exposure and dissociation. For one thing, people may fear that their privatized bodies will be publicly exhibited to strangers when procuring organs. The introverted and self-restrained characteristics of the Eastern culture further consolidate this perception. For another, the mutilated and disfigured body imagery becomes a mental disturbance. In a similar vein, the previous study has identified the fear of disruption of body integrity as one principal reason for refusing organ donation (49, 50). Additionally, the traditional Chinese values, such as “maintain an intact body after death,” also subconsciously keep people away from body dissociation operations.

*Suddenly, I was so scared! I don't know why I'm so sensitive to life and death! I don't want to donate my organs, nor my body! Because I still want to keep myself intact, no matter now or later*.

*Every time I look at the tomographic specimens, the thought of donating organs comes to my mind. The passion is always accompanied by intense terror. I don't know whether the fear of death or the feudal education engraved in the bone makes me scared of being sliced after death without a soul*.

#### Familial level: Opposition from family members

In contrast to the previous study, 58.13% of the disapproval comes from family members' opposition, which is the most significant perceived barrier in our study. Regardless of country, a large percentage of organ donation decisions are not made by the donor per se but rather by the family members after the donor's death. In China, if a citizen does not expressly disagree during his or her lifetime, the immediate family of that citizen has a 100% right to decide on organ donation after his or her death (51). Families have an inherently influential, if not decisive, position in organ donation. We provide two examples of posts.

*Dad said, “We are both highly educated, and we can accept this (organ donation) from a rational point of view. But we are not alone. We all have relatives. We have to consider the feelings of our family members. Let's imagine one of your family members who was breathing and alive at this moment, but in the next second, he or she passed away. A group of people removes all the available organs immediately. What do you think of it?”*

*I asked my mother for her opinion on signing the donation petition, she disagreed. When I went to the Red Cross to apply, the staff told me that if your husband or your immediate family members objected, you would not be able to donate your organs. I was furious, and I said, “Shouldn't my own will take precedence?”*

The family-related discourse about the organ donation topic is particularly pronounced in China. Liu accentuated that Chinese society has a nature of consanguinity (or the so-called kinship) rather than individualism or collectivism (52), which means that family is given priority in this particular cultural context (19). As a culturally embedded persuasive force, filial piety shapes the behavioral principle in every Chinese family (53). To comply with filial piety, Chinese people are inclined to adhere to the norm that “the body and the skin are gifts from parents.” Therefore, misunderstanding and opposition from family members become a salient barrier to organ donation in China.

More importantly, the opposition from family is interspersed with other social representation elements. Family concerns permeate into how the individual contemplates death. Family traditions or disciplines also echo the common cultural roots of Chinese people. Thus, the conversations about organ donation with family members are often related to spiritual beliefs. We offer some examples below.

*I saw a blogger posting about registering for organ donation. I registered a few days ago, but I was afraid to mention it to my parents. Although they occasionally watch programs about organ donation on TV, I don't know what kind of attitude they hold toward it. I worry that they would be reluctant to talk about it because of their taboo against “death.”*

*I received the registration card for the organ donation volunteer. My mother saw it with a huge shock! She immediately blamed me and reminded me that it is inauspicious to consider organ donation at such a young age. “You must be crazy! That means no whole corpse!” I am so easily influenced by my mom. Now I am stuck in a dilemma*.

#### Societal level: Distrust toward medical institutions and society

This category occupies 7.03% of the whole filtered corpus, a relatively small percentage compared to those studies carried out in Western societies (54, 55). Consistent with previous studies, denial and rejection of brain death hindered the decision to donate organs (56), which in turn triggered worries about the early termination of medical treatment and inadequate care for donors. All of those concerns bring about mistrust toward hospitals. There also exist posts oppugning the integrity of the healthcare system, fairness of the organ allocation procedure, and transparency of the double-blind design. For instance, some people fear that their organs may be brutally removed, or their organs may be supplied to the powerful class or evil person.

*My reason for the reluctance to donate organs is quite simple: what if the person who gets my organs is not a good guy? What if the person who survives brings misfortune to other people?*

*One day in the future, if the opportunity to be a recipient is genuinely equal, with no money or power involved, I would donate without hesitation*.

*I thought that I would like to register for organ donation. But I'm afraid of information leakage. Someone may kill me to get my organs*.

#### Cultural level: Religious-cultural concerns about fatalism

This category accounts for 14.06% of the overall obstacles in organ donation. According to Chinese tradition, the concepts of rebirth and ancestor worship challenge the implementation of organ donation (16, 57). Chinese people believe in keeping their organs and bodies intact in anticipation of being reborn as human beings in the next samsara. Otherwise, they may be abandoned in the reincarnation process (58). Despite the religious shift among the Chinese in recent years, the above spiritual, or even superstitious notions, continued to exert influences on public perceptions. We provide two typical narratives below.

*I have been thinking about the morality and Buddhist laws of organ transplantation and donation. It is beyond doubt that donating a body after death is a noble virtue and colossal support to scientific research. But in Buddhist tenets, you cannot do it because it would lead to falling into hell*.

*The more I grew up, the more superstitious I became … I am afraid of many unknown things … Donate or not, not because of other people's opinions, but to the reflection of the meaning of it to my life*.

## Discussion

### Principal findings

Organ donation has always been highlighted in the public health agenda, intriguing public and scholarly attention. To our knowledge, this study is among the few to address the organ donation issue in China by exploiting natural expressions on social media. Following a corpus-driven approach, we disentangled from predefined theoretical frameworks and allowed the emergence of perceptions and perceived barriers. Previous studies examined the antecedents of organ donation statistically (59, 60) but lacked profound inquiry into the whole opinion landscape during an extended period. To supplement, we jumped out of a specific time phase and inspected the general trend of social representation from 2010 to 2020. Firstly, it can be observed distinctly from Figure 1 that organ donation discussed by ordinary users corresponds to the trend of total posts, with an evident surge after 2018. This sudden surge has been impacted chiefly by policy guidance. The *Law of the People's Republic of China on Red Cross Society* was amended in May 2017 to legitimize organ donation, which clarified the responsible agency for organ donation. In March 2018, the Human Organ Donation Management Center launched a nationwide campaign to memorize human organ donors, stirring large-scale public discussion. In a nutshell, the national agenda successfully led the public's agenda, even for sensitive topics like organ donation.

Secondly, this work enriches the study of the social representations of organ donation in the Chinese context. In conformity with Liu as well as Moloney and Walker, we back up the coexistence of antithetical representations (5, 8, 52). The longing for survival and the fear of death were juxtaposed to create a dialectical perception prevailing in China. On the one hand, it is conspicuous that many people intended to pass hope or great love to others or even aspired to contribute to pushing forward medical research by donating their organs. On the other hand, the desire for a decent death, along with the ingrained reverence for life, counterbalance the dedication. The two forces intertwined and maintained an equilibrium. This pair of antinomic representations shape the primary psychological state of potential Chinese donors.

Thirdly, the family-related narratives are dispersed over multiple representations, including “hope, understanding, and acceptance,” “family story,” and “the practical value of organ donation.” What's more, hindrance from the familial level predominates all the barriers. Hence, family support is of paramount importance to organ donation in China. Not only because the family unit was the cornerstone that constitutes the conventional Chinese society, but also because the family culture renders the bottom color of the Chinese culture.

Fourthly, the Chinese cultural context was examined from a unique perspective. Although the dilemma of reluctance to organ donation troubles the whole world, the Chinese esoteric attitude toward organ donation deserves further exploration. To a certain extent, impacted by cultural traditions such as Confucianism, Buddhism, and Taoism, Chinese society is not so supportive of cadaveric organ donation (61). Some scholars found that the organ donation system in China is far behind the international level (59, 62). One probable explanation can be attributed to the deeply embedded traditions about the significance of good death in Chinese society (61, 63). Dutta put forward the cultural sensitivity approach to underscore the crucial position of cultural characteristics in health interventions (64). Our concentration on the Chinese cultural context somewhat dovetails this approach and reminds latecomers of the importance of cultural traits when probing into organ donation.

Lastly, the current endeavor established a framework concerning organ donation's hindrances, casting light on future empirical studies. Prior analyses on organ donation intention were always grounded on individual-level behavioral theories and focused on limited motivating or obstructive factors (19, 65, 66). We extended the impediments to four layers, encompassing individual, familial, societal, and cultural factors. Informed by the proposed hierarchical model, as shown in Figure 3, scholars could adapt existing measures or develop new scales to cover all the essential constructs to better grasp what inhibits potential donors from performing the actual behavior. For example, beyond self-efficacy and perceived social norms, public health pundits need to allocate attention to pressures from parents and the broader cultural context. Furthermore, our framework contributes to comparative studies. Some extant studies have attempted to understand health-related behavior disparities in cross-cultural settings (67, 68). By following our systematic model, follow-up studies can distinguish what factors are more influential in affecting organ donation in other cultures. Findings from the comparative perspective would undoubtedly facilitate organ donation worldwide and inform the designing of effective persuasive messages on organ donation.

### Practical implications

Based on the four levels distilled from the conventional content analysis. We propose the following strategies to promote organ donation in China for public health pundits. Firstly, at the individual level, more public health education is needed to enhance the Chinese public's knowledge level regarding organ donation. Since organ donation is a sensitive topic in China, the government and public institutions should lead the tide in desensitizing organ donation by encouraging more people to learn the scientific principles and operation process behind organ donation. For example, in 2022, Zhejiang Province in China took the lead in incorporating organ donation knowledge into textbooks, which helped dispel the mystery of organ donation (69). Secondly, at the familial level, family-unit-based health intervention is an urgent need because family members' disapproval is one of the most significant barriers. Moreover, the family-related representations even bridge the individual and sociocultural levels and permeate the whole collection of barriers. Therefore, the government could take advantage of the family unit to persuade organ donation and allocate more attention to the interaction of family members. For example, the UK NHS (United Kingdom National Health Service) launched a campaign in 2021 to facilitate discussions among two generations regarding organ donation. Effects of the campaign proved family intervention's effectiveness in creating a supportive family atmosphere that benefits positive attitudes toward organ donation (70). China can borrow this experience. Thirdly, at the societal level, although distrust toward medical institutions and the whole society is not that significant compared to other barriers, the government can devote itself to enhancing public confidence and trust in public institutions. For instance, the China Organ Transplant Response System (COTRS)—a system that was progressively introduced in China to make the organ donation and allocation process more transparent (71), could be a promising way to quell the doubts of the public. Fourthly, at the cultural level, the transformation of religious-cultural concerns is a long process, which necessitates a joint effort from all societal sectors. The individual should absorb more evidence-based information regarding modern medical technologies like organ donation to counterbalance cultural concerns. Meanwhile, the government needs to advocate a new social ethos to limit the expansion of superstitious beliefs. A typical example is the Healthy China Initiative, which promotes medical and scientific information in ordinary communities across China to encourage evidence-based decisions and critical thinking (72). Future public health campaigns should proceed with this endeavor for a new social climate.

Furthermore, our study adopts an unobtrusive way to excavate the social media platform for social representations regarding public health issues. The findings advance our understanding of how people perceive organ donation over a relatively long period. This manner of scrutinizing digital traces outcompetes the traditional social survey or in-depth interview, for they can hardly unfold long-term public perceptions or perception fluctuation in a longitudinal sense. In concordance with preceding works on vaccination perception (31), emerging infectious diseases (73), and chronic diseases (74), the current study bolsters the idea that public health researchers should take full advantage of social media to comprehend how the public makes sense of some vital health issues. Moreover, temporal and spatial dimensions can be integrated for a fine-grained dataset, enabling scholars to answer more intricate questions, such as how significant social events disturb an established social representation toward organ donation? Did social representations about organ donation vary across provinces or states?

## Limitations

Our retrospective observational study has some limitations. The Chinese public was approximately substituted by the general public user group on Weibo, which is a compromise suffering validity threats because there exist some Chinese who do not use social media or have no access to social media. Besides, scholars have cautioned that “digital footprints left behind by technology users are rarely representative” (44). Therefore, we should be keenly aware of the biased nature of the current dataset (e.g., the unbalanced distribution of age, gender, occupation, or other demographic characteristics), which implies that conclusions drawn from this work cannot be easily generalized to the entire population. It is necessary for future researchers to cross-validate our findings by conducting national surveys based on probabilistic sampling strategies. Furthermore, since the research corpus covers 11 years, some momentous events may change how people perceive organ donation. The possible changes across the years are open to further empirical testimony.

## Conclusion

This study utilized a hybrid text analysis approach on social media corpus to excavate the Chinese public's social representations of organ donation and perceived barriers over the past decade. Five pivotal representations were distilled, and a four-layer hierarchical model regarding hindrances was proposed to understand public perceptions toward organ donation in China.

## Data availability statement

The original contributions presented in the study are included in the article/Supplementary material, further inquiries can be directed to the corresponding author.

## Author contributions

ZZ and JJ: conceptualization, formal analysis, methodology, project administration, visualization, writing—original draft, and writing—review and editing. CL: conceptualization, formal analysis, methodology, writing—original draft, and writing—review and editing. AC: conceptualization, data curation, and writing—review and editing. All authors contributed to the article and approved the submitted version.

## Conflict of interest

The authors declare that the research was conducted in the absence of any commercial or financial relationships that could be construed as a potential conflict of interest.

## Publisher's note

All claims expressed in this article are solely those of the authors and do not necessarily represent those of their affiliated organizations, or those of the publisher, the editors and the reviewers. Any product that may be evaluated in this article, or claim that may be made by its manufacturer, is not guaranteed or endorsed by the publisher.
