# Supplementary material for: Excavating the social representations and perceived barriers of organ donation in China over the past decade: A hybrid text analysis approach
Source: Front Public Health. 2022 Sep 26;10:998737. doi: 10.3389/fpubh.2022.998737 (PMC9549352; doi:10.3389/fpubh.2022.998737)
Supplement: Supplementary file 1 [file Data_Sheet_1.docx]

| **ID** | **Search term in Chinese** | **Search term in English** |
| --- | --- | --- |
| 1 | 器官捐献 | Organ donation |
| 2 | 捐器官 | Donate organs |
| 3 | 捐献器官 | *Ibid.* |
| 4 | 遗体捐献 | Body donation |
| 5 | 捐遗体 | Donate the body |
| 6 | 捐献遗体 | *Ibid.* |
| 7 | 捐肾 | Donate kidney |
| 8 | 捐肝 | Donate liver |
| 9 | 捐心 | Donate heart |
| 10 | 捐胰腺 | Donate pancreas |
| 11 | 捐肺 | Donate lung |
| 12 | 捐小肠 | Donate small intestine |
| 13 | 捐角膜 | Donate cornea |
| 14 | 捐眼角膜 | *Ibid.* |
| 15 | 捐骨髓 | Donate marrow |

***Supplementary Material A: Bilingual inventory of search terms***
