# Supplementary material for: Excavating the social representations and perceived barriers of organ donation in China over the past decade: A hybrid text analysis approach
Source: Front Public Health. 2022 Sep 26;10:998737. doi: 10.3389/fpubh.2022.998737 (PMC9549352; doi:10.3389/fpubh.2022.998737)
Supplement: Supplementary file 2 [file Data_Sheet_2.docx]

***Supplementary Material B: Semantic networks and social representations summaries based on each year’s corpus***


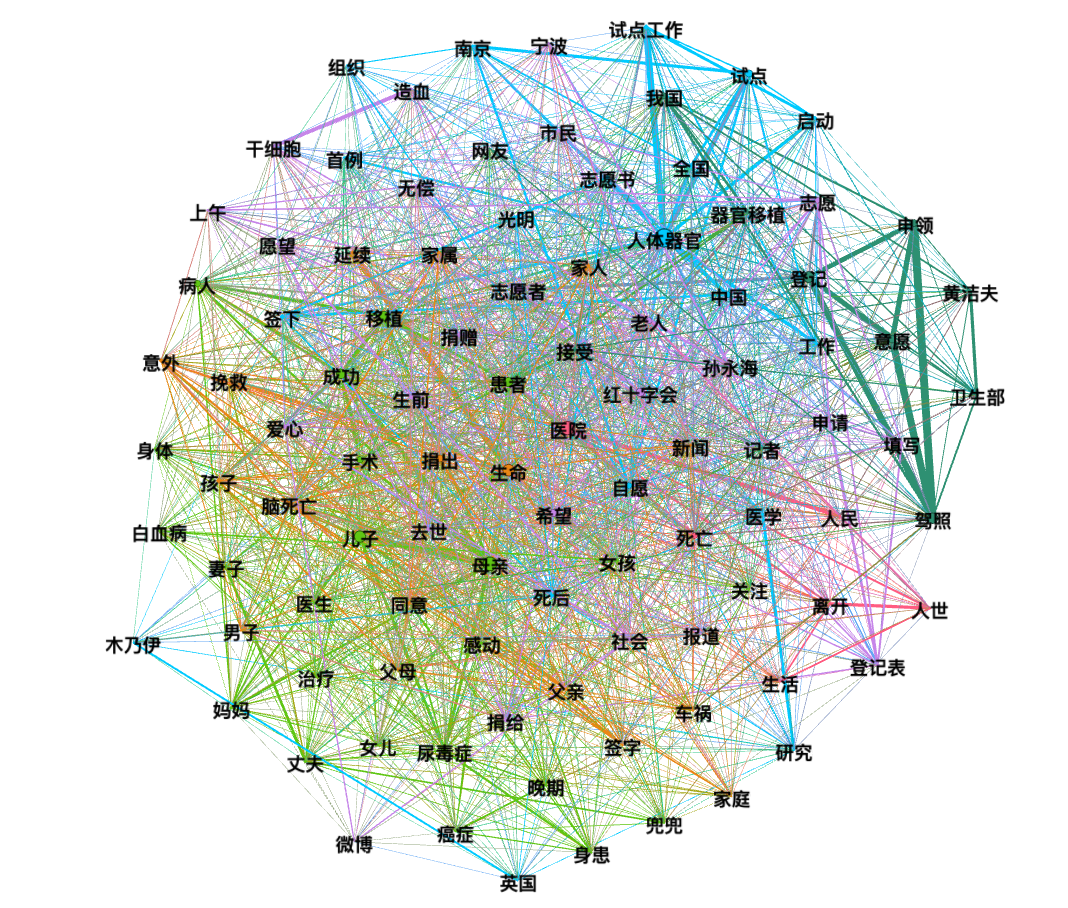


Figure 1 Visualization of the semantic network regarding organ donation, from 2010 to 2011


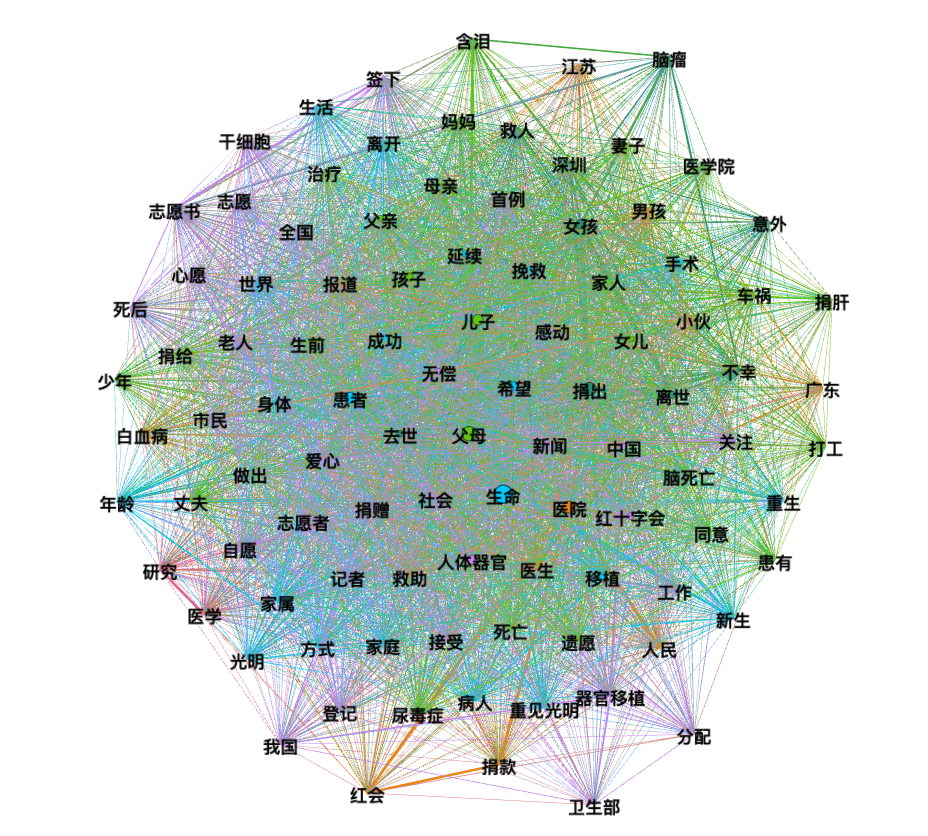


Figure 2 Visualization of the semantic network regarding organ donation, from 2012 to 2013


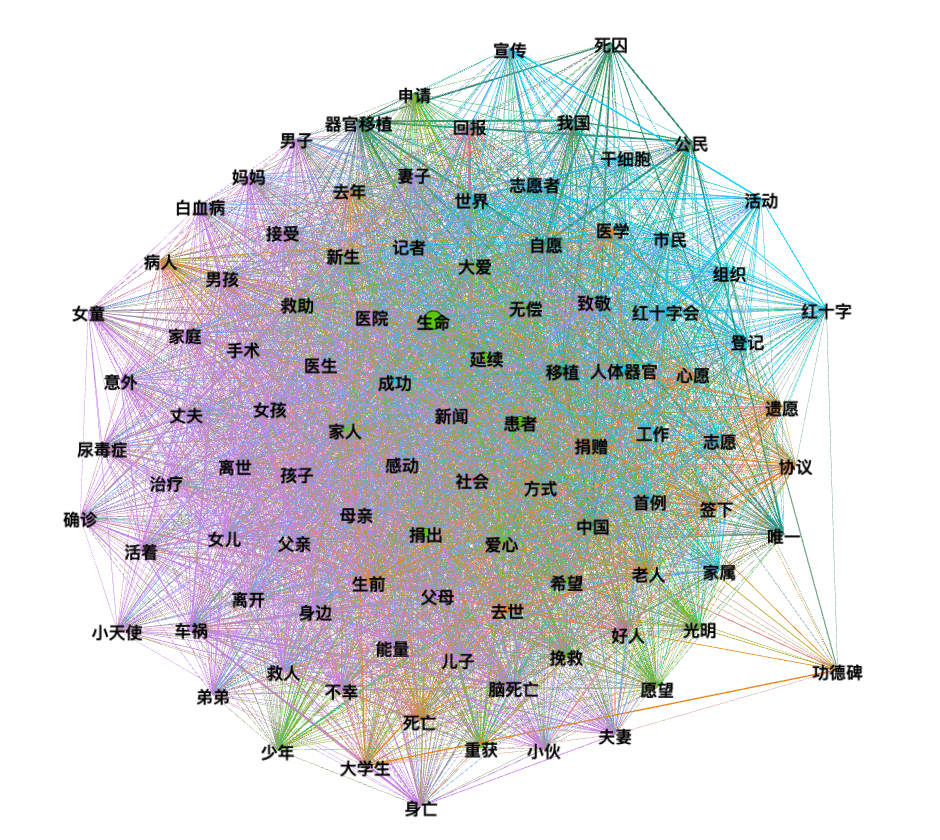


Figure 3 Visualization of the semantic network regarding organ donation, from 2014 to 2015


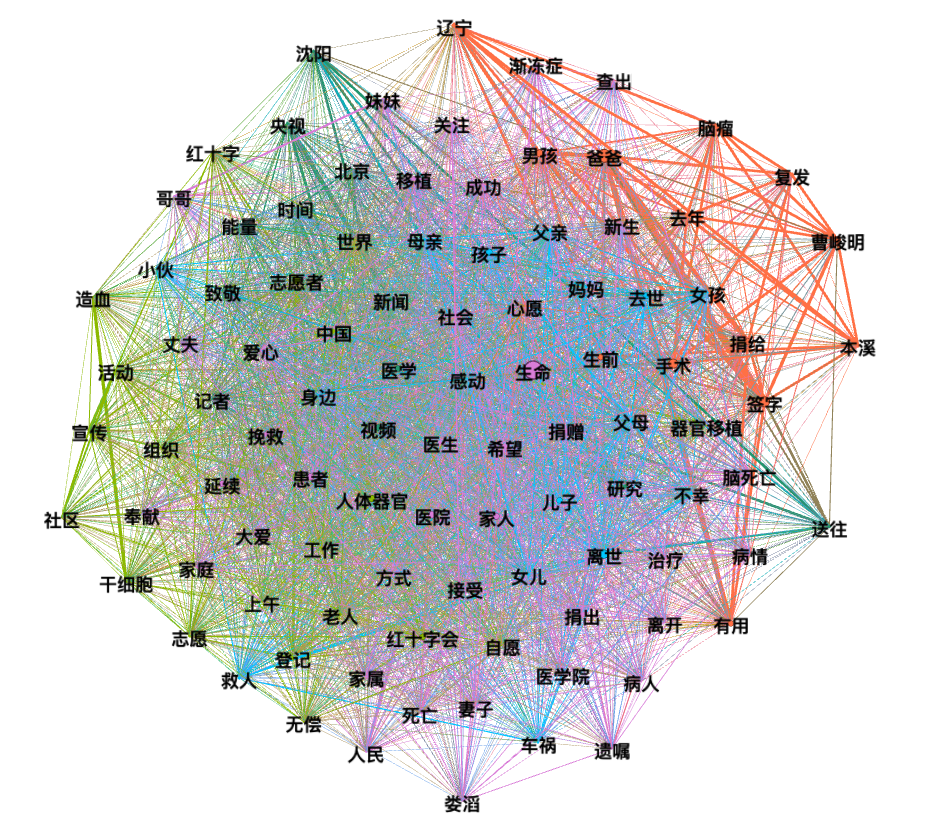


Figure 4 Visualization of the semantic network regarding organ donation, from 2016 to 2017


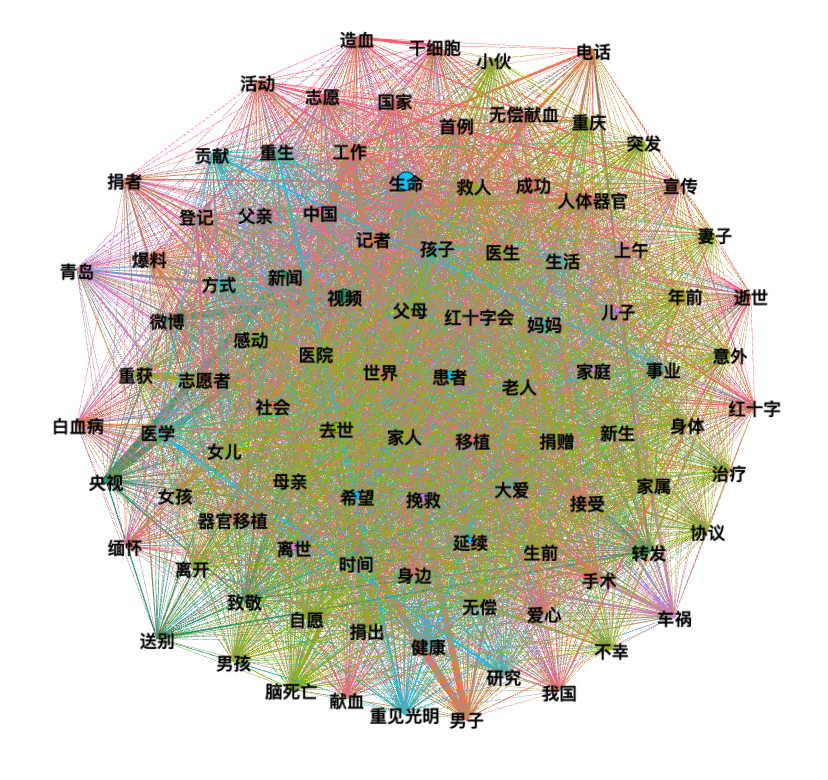


Figure 5 Visualization of the semantic network regarding organ donation, from 2018 to 2019


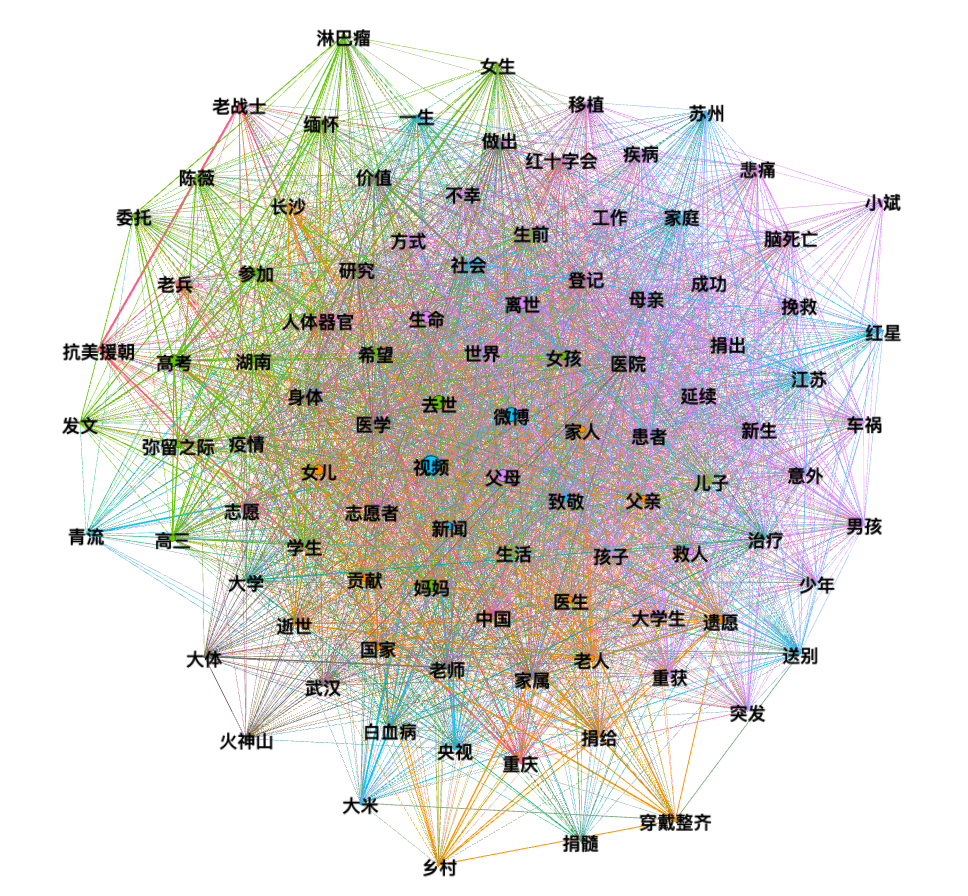


Figure 6 Visualization of the semantic network regarding organ donation in 2020

**Table 1 Social representations summary of organ donation over 11 years**

| period | counts | No. | percentage | representation | keywords |
| --- | --- | --- | --- | --- | --- |
| 2010-2011 | 152 | 1 | 11% | governmental discourse | Huang Jiefu; application |
|  |  | 2 | 17% | the practical value of organ donation | brain death; donation |
|  |  | 3 | 6% | death and life | death; departure |
|  |  | 4 | 19% | policy about organ donation | polit; volunteer |
|  |  | 5 | 23% | family story | mother; husband |
|  |  | 6 | 29% | the procedure of organ donation in NGO | Red Cross |
| 2012-2013 | 1120 | 1 | 21% | death and life | life; hope |
|  |  | 2 | 30% | the procedure of organ donation in NGO | Red Cross; volunteer |
|  |  | 3 | 9% | specific event | Shenzhen; girl |
|  |  | 4 | 26% | family story | parents; father |
|  |  | 5 | 12% | organ donation in the hospital | hospital; doctor |
|  |  | 6 | 2% | organ donation about medication | medication; research |
| 2014-2015 | 596 | 1 | 40% | family story | parents; father |
|  |  | 2 | 14% | death and life | pass away; before death |
|  |  | 3 | 3% | the practical value of organ donation | society; reward |
|  |  | 4 | 17% | the procedure of organ donation in NGO | volunteer; Red Cross |
|  |  | 5 | 8% | policy about organ donation | transplant; death row |
|  |  | 6 | 18% | discourse about life | patient; hope |
| 2016-2017 | 747 | 1 | 14% | specific event | Cao Junming; brain tumor |
|  |  | 2 | 12% | organ donation about medication | video; medication |
|  |  | 3 | 20% | the procedure of organ donation in NGO | volunteer; Red Cross |
|  |  | 4 | 16% | family story | child; parents |
|  |  | 5 | 38% | the practical value of organ donation | life; love |
| 2018-2019 | 75791 | 1 | 8% | the practical value of organ donation | salute; CCTV |
|  |  | 2 | 16% | discourse about life | patient; hope |
|  |  | 3 | 5% | specific event | man; work |
|  |  | 4 | 8% | family story | save; family |
|  |  | 5 | 32% | discourse about death | pass away; before death |
|  |  | 6 | 29% | the procedure of organ donation in NGO | volunteer; Red Cross |
| 2020 | 36966 | 1 | 14% | the practical value of organ donation | Weibo; salute |
|  |  | 2 | 10% | specific event | veteran |
|  |  | 3 | 28% | death and life | pass away; being |
|  |  | 4 | 19% | specific event | mom; college entrance examination |
|  |  | 5 | 13% | specific event | girl; country teacher |
|  |  | 6 | 12% | organ donation about medication | research |
|  |  | 7 | 4% | others | university; leukemia |
