# Supplementary material for: Excavating the social representations and perceived barriers of organ donation in China over the past decade: A hybrid text analysis approach
Source: Front Public Health. 2022 Sep 26;10:998737. doi: 10.3389/fpubh.2022.998737 (PMC9549352; doi:10.3389/fpubh.2022.998737)
Supplement: Supplementary file 3 [file Data_Sheet_3.docx]

***Supplementary Material C: The complete semantic network without edge weight filtration***


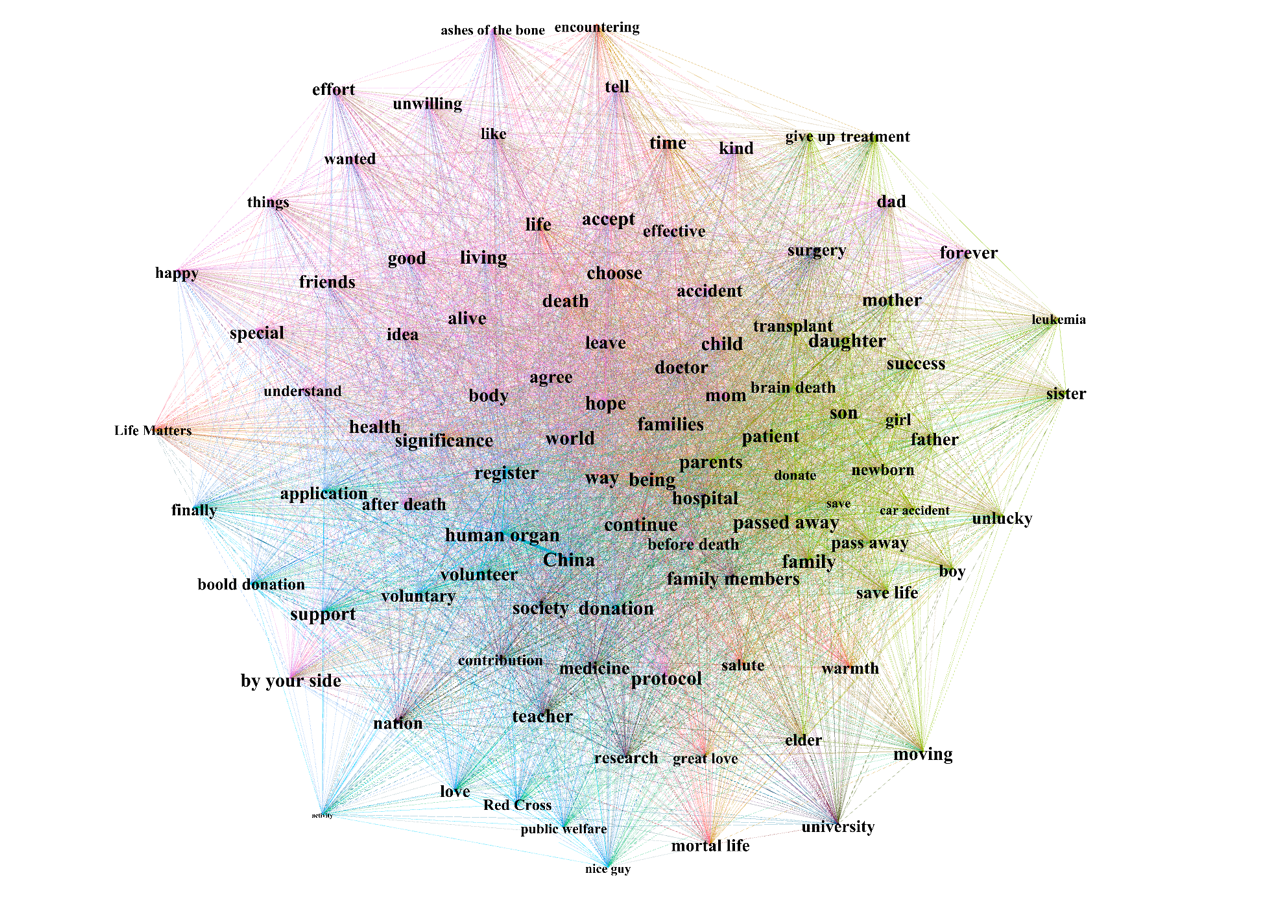


Figure 1 The complete semantic network of organ donation, from 2010 to 2020
