# Supplementary material for: Excavating the social representations and perceived barriers of organ donation in China over the past decade: A hybrid text analysis approach
Source: Front Public Health. 2022 Sep 26;10:998737. doi: 10.3389/fpubh.2022.998737 (PMC9549352; doi:10.3389/fpubh.2022.998737)
Supplement: Supplementary file 4 [file Data_Sheet_4.docx]

***Supplementary Material D: Training and Output of the LDA topic model***


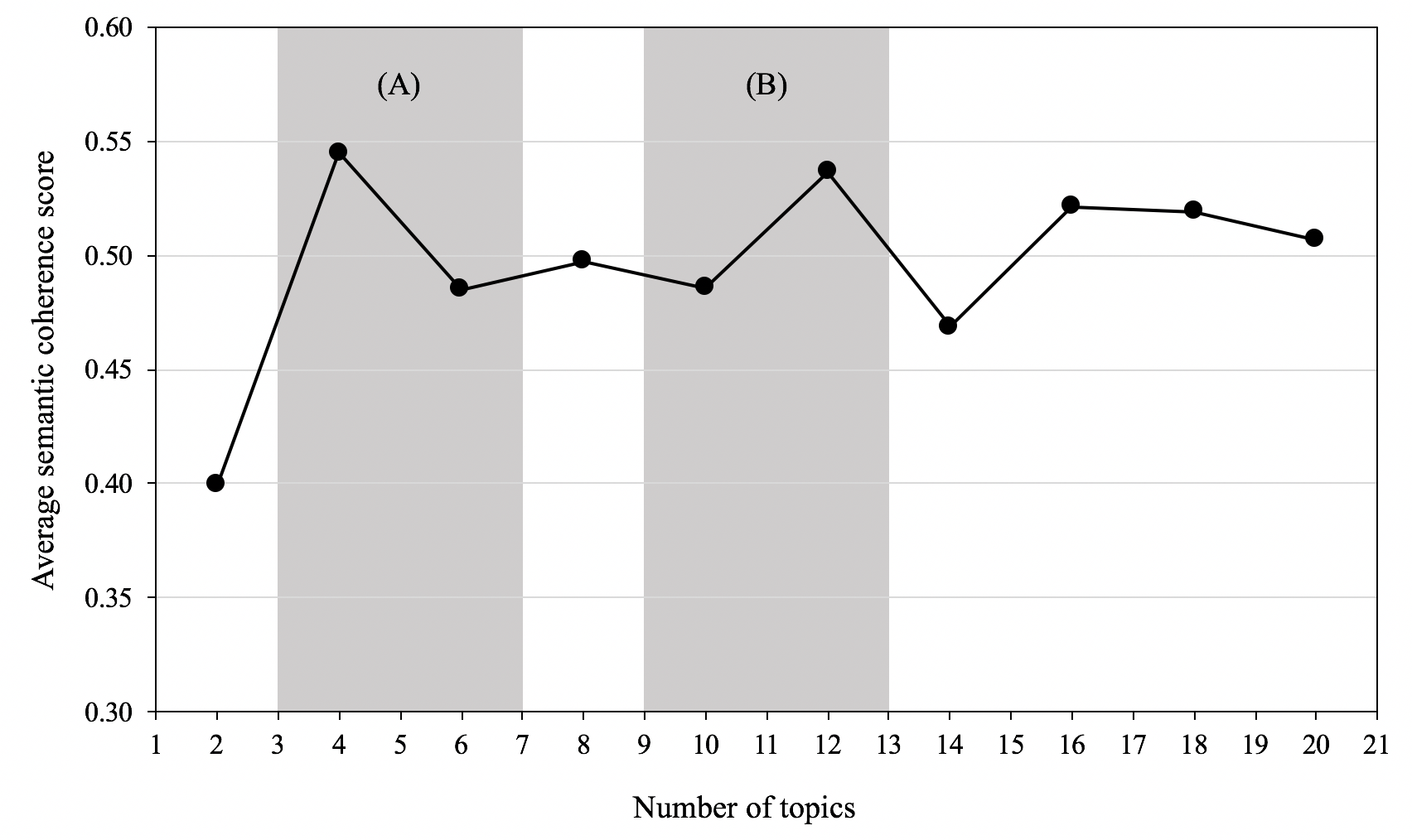


**Figure 1 Average semantic coherence score under different numbers of topics.**

*Note.* We conducted a grid search to choose the best hyper-parameter combination for our LDA-based topic model. A 10 (number of topics (k), ranges from 2 to 20 with an interval of 2) * 2 (alpha, set as symmetric or asymmetric) * 2 (beta, set as symmetric or asymmetric) = 40 combinations were tested. The average semantic coherence score was calculated by taking the mean value of all scores under each number of topics. According to the score fluctuation, we built candidate topic models (in area (A), number of topics = 3, 4, 5, 6, 7. In area (B), number of topics = 9, 10, 11, 12, 13). Taking interpretability as the prime criterion in selecting models, we choose k = 5 as the final topic parameter.

**Table 1 Output of LDA topic modelling.**

| NO. | percentage | Top 10 keywords |
| --- | --- | --- |
| 1 | 28% | society; China; human organ; volunteer; register; being; volunteering; Red Cross; activity; love |
| 2 | 23% | hope; being; world; living; child; mother; signification; life; Life Matters; kind |
| 3 | 13% | doctor; hospital; medication; family member; surgery; patient; elder; research; being; salute |
| 4 | 25% | death; register; agree; unwilling; body; ashes of bone; after death; teacher; fear; parents |
| 5 | 12% | pass away; girl; being; passed away; save life; mom; newborn; news; car accident; donation |
